# Effects of low-FODMAP diet on irritable bowel symptoms in patients with quiescent inflammatory bowel disease: A protocol for a systematic review and meta-analysis

**DOI:** 10.1097/MD.0000000000029088

**Published:** 2022-03-18

**Authors:** Baijian Gu, Zhe Yu, Chong Shi, Chengqiu Yan, Bixin Chen, Jianhua Zhou

**Affiliations:** ^a^ *Changchun University of Chinese Medicine, Changchun City, Jilin Province, China,* ^b^ *Urology, First Affiliated Hospital to Changchun University of Chinese Medicine, Changchun City, Jilin Province, China,* ^c^ *Proctology, First Affiliated Hospital to Changchun University of Chinese Medicine, Changchun City, Jilin Province, China,* ^d^ *Electronic data processing management science, Health Information Center of Jilin Province, Changchun City, Jilin, China.*

**Keywords:** Crohn disease, inflammatory bowel disease, low-FODMAP diet, protocol, safety, systematic review, ulcerative colitis

## Abstract

**Background:**

Inflammatory bowel disease (IBD) is a chronic disease whose etiology is not yet fully understood, and their course is characterized by periods of exacerbation and remission. In quite a few cases, actual disease remission may also accompany with inflammatory bowel disease (IBS)-like symptoms such as abdominal pain, bloating, flatulence, and diarrhea, may greatly impact quality of life. An army of strong evidence to support the FODMAPs diet (LFD) compounds as an effective dietary approach to IBS treatment. However, there is no significant evidence showing the effectiveness of LFD in treating quiescent IBD and its side effects; this lack of evidence is also an important factor hindering its promotion in the treatment of IBD and its complications. Therefore, this systematic review and meta-analysis will evaluate the efficacy and safety of LFD in the treatment of quiescent IBD patients with IBS-like symptoms.

**Method:**

We searched the following databases from their establishment until December 2021: PubMed, Web of Science, Embase, Cochrane Library, CNKI, VIP, and Wanfang databases. No restrictions regarding publication date or language were applied. Keywords such as “Crohn’s disease,” “ulcerative colitis,” “inflammatory bowel disease,” and “FODMAPs” have been combined for search. Ongoing and unpublished research in the Clinical Trials Registry Research will also be included. At the same time, we will manually search all reference lists from relevant systematic reviews for other eligible studies. The selected studies were randomized controlled clinical trials. We will meta-analyze the selected literature by Review Manager software (REVMAN v5.4 Cochrane Collaboration). Two researchers will independently review the research selection, data extraction, and research quality assessments. Finally, we will observe the outcome measures.

**Results:**

This study will provide evidence-based data for TFD treatment of IBD and provide new treatment options for future clinical applications.

**Ethics and dissemination:**

The protocol of the systematic review does not require ethical approval because it does not involve humans. This article will be published in peer-reviewed journals and presented at relevant conferences.

**Registration Number:**

INPLASY202220060

## 1. Introduction

Inflammatory bowel disease (IBD) includes Crohn diseaseand ulcerative colitis (UC), both of which have similar symptoms of digestive disorders and inflammation of the digestive system.^[1]^ IBD is common in Western countries, particularly in northern Europe and North America. Approximately 1.6 million Americans are suffering from IBD, with 785,000 patients with CD, and 910,000 with UC.^[2]^ Although there was no significant sex difference in the prevalence of the 2 diseases, the age of onset of CD was slightly earlier, with an average age of 15 to 25 years, whereas the age of onset of UC patients was between 25 and 35 years.^[3]^ IBD is a chronic disease whose etiology is not yet fully understood, and their course is characterized by periods of exacerbation and remission. In IBD patients, if intestinal inflammation is controlled and there are no active clinical symptoms, the disease is in remission. There are 4 levels of remission of IBD, including stable disease, clinical, biological indicators, and endoscopic remission. In many cases, actual disease remission may not be accompanied by complete resolution of symptoms, as patients in remission or with minimal disease activities. The etiology of these intestinal symptoms in quiescent IBD is also lies in the mist, but is presumed to be associated with coexisting IBS, the impact of exacerbated gastrointestinal inflammation on bowel function, long-term unclear low-grade inflammation, or psychological impact on IBD relapse.^[4]^ The FODMAPs diet (LFD) includes fructose, lactose, fructo- and galacto-oligosaccharides (fructans and galactose), and polyols, which are poorly absorbed in the small intestine and are passed to the colon where they act osmotically to draw fluid into the lumen.^[5]^ In addition, leading to an increase in gas production (mainly methane and hydrogen) due to excess transport of fermentative substrates to the colonic microflora. These pathologies are often caused irritable bowel symptoms (IBS)-like symptoms such as abdominal pain, bloating, flatulence and diarrhea, may greatly impact quality of life.^[6]^ A lot of strong evidence^[7-9]^ to support LFD compounds as an effective dietary approach to IBS treatment. However, there is no significant evidence showing the effectiveness of LFD in treating quiescent IBD and its side effects; this lack of evidence is also an important factor hindering its promotion in the treatment of IBD and its complications. Therefore, this systematic review and meta-analysis will evaluate the efficacy and safety of Low-FODMAP diet in the treatment of quiescent IBD patients with IBS-like symptoms.

## 2. Materials and methods

### 2.1. Information sources and search strategy

This study will be based on the reporting guidelines of the Protocols and Meta-Analysis of Systematic Reviews (PRISMAP).^[10]^ We conducted this meta-analysis using previously published studies; no patients were involved in this study; therefore, no informed patient’s consent and/or public ethical approval were required. We searched the following databases from their establishment until December 2021: PubMed, Web of Science, Embase, Cochrane Library, CNKI, and Wanfang databases. No restrictions regarding publication date or language were applied. Keywords such as “Crohn’s disease,” “ulcerative colitis,” “inflammatory bowel disease,” and “FODMAPs” have been combined for search and the search strategy in PubMed is shown in Table 1. Ongoing and unpublished research in the Clinical Trials Registry Research will also be included. At the same time, we will manually search all reference lists from relevant systematic reviews for other eligible studies. The study has been approved by the International Platform for Registration System Review and Meta-Analysis Protocol. (https://inplasy.com/inplasy-2022-2-0060/). The registration number is INPLASY202220060.

**Table 1:** Search strategy for the PubMed database.

**Number**	**Terms**
#1	Crohn Disease(all fifield)
#2	Crohn’s Enteritis(all fifield)
#3	Regional Enteritis(all fifield)
#4	Crohn’s Disease(all fifield)
#5	Inflammatory Bowel Disease 1(all fifield)
#6	Granulomatous Enteritis(all fifield)
#7	Enteritis, Regional(all fifield)
#8	colitis, ulcerative(all fifield)
#9	idiopathic proctocolitis(all fifield)
#10	ulcerative colitis(all fifield)
#11	colitis gravis(all fifield)
#12	inflammatory bowel disease, ulcerative colitis type(all fifield)
#13	Inflammatory Bowel Disease(all fifield)
#14	Bowel Diseases, Inflammatory(all fifield)
#15	#1 or #2-14
#16	FODMAP (all fifield)
#17	FODMAPs(all fifield)
#18	fermentable oligosaccharides, disaccharides and monosaccharides and polyols(all fifield)
#19	fermentable, poorly absorbed, short chain carbohydrates(all fifield)
#20	#16 or #17-19
#21	randomized controlled trial (all fifield)
#22	randomly (all fifield)
#23	controlled clinical trial (all fifield)
#24	randomized (all fifield)
#25	random allocation (all fifield)
#26	placebo (all fifield)
#27	single-blind method (all fifield)
#28	double-blind method (all fifield)
#29	trials (all fifield)
#30	comparators
#31	allocation
#32	#21 or #22-31
#33	#15 And #20 And #32

### 2.2. Inclusion and exclusion criteria

The inclusion criteria for the literature search will be as follows: studies including patients aged ≥ 18 years, regardless of sex, with IBD diagnosed according to the standards of the American Gastrointestinal Association or the European Society for Gastrointestinal Endoscopy; randomized controlled studies; studies with LFD in the experimental group and controls group was on a normal diet. The exclusion criteria will be as follows: studies including patients aged < 18, IBD in exacerbation; patients without IBS-like symptoms; studies in which the experimental group was given treatment methods other than Low-FODMAP diet; studies in which the control group was not only treated with normal diet; studies that were not randomized controlled trials.

### 2.3. Study selection

Two researchers independently conducted literature searches; search strategies and data extraction tables were based on preestablished criteria; nonclinical studies such as conference proceedings, newspapers, and guidelines would be excluded. A PRISMA flowchart^[11]^ will be designed to illustrate the study selection process. If an article is missing the full text or data necessary for the analysis, we will contact the article authors and request relevant data. The 2 independent researchers assessed the quality of the included studies according to the Cochrane Manual Handbook^[12]^ independently and then reported the results according to the PRISMA guidelines.^[13]^ Any disagreement will be resolved by discussion until consensus is reached, if divisions persisted, then the third investigator will be consulted to make the final and irrevocable decision. A flowchart of the screening process is shown in Figure 1.

**Figure:**
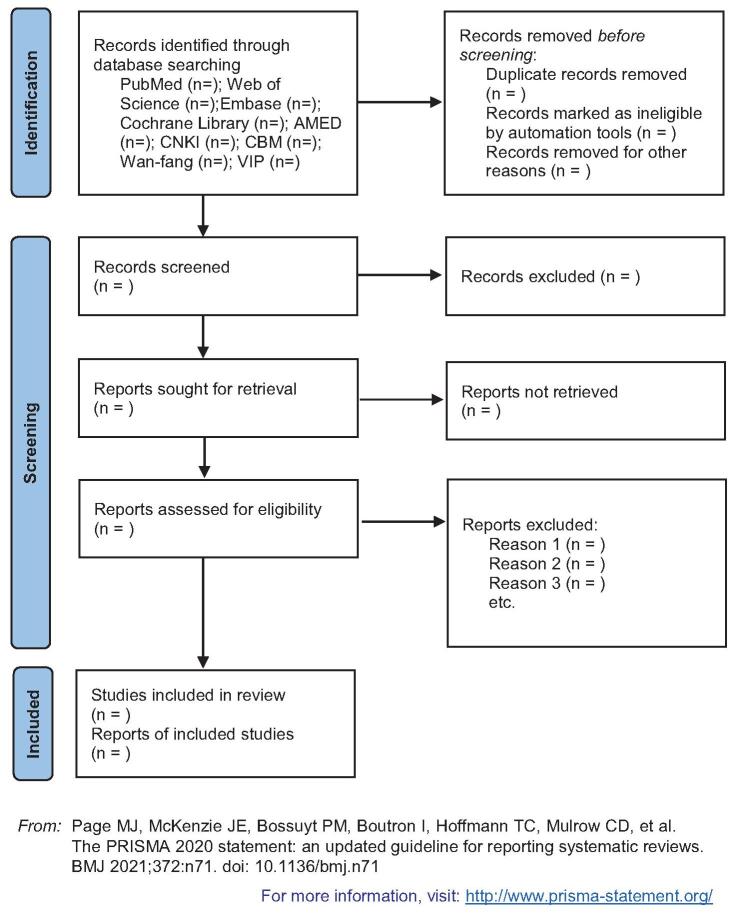
**Figure 1.** Flow diagram of study selection process.

### 2.4. Assessment of study quality

Two investigators will separately assess the risk of bias of the selected RCTs by the Cochrane risk of bias assessment tool.^[14]^ It consists 7 items: random sequence generation, allocation concealment, blinding of participants and personnel, blinding of outcome assessment, incomplete outcome data, selective reporting, and other bias. We will use Begg and Egger tests and set *P* < .1 as statistically significant, and we will use a funnel chart to assess publication bias. These even domains will be separately appraised by 2 reviews, and discrepancies will be addressed by consulting a third reviewer.

### 2.5. Outcome measures

The primary outcome measures will include the gut symptoms, patient’ quality of life, and disease activity. The secondary outcome measures will include inflammatory markers fecal calprotectin. Adverse events resulting from safety violations will be reported.

### 2.6. Statistical analysis

We will meta-analyze the selected literature by Review Manager software (REVMAN v5.4 Cochrane Collaboration), and *P* < .05 was considered statistically significant. The risk ratio and 95% confidence intervals (CIs) were collected for enumeration data, while the mean difference or standardized mean difference and 95% confidence intervals were used to calculate continuous outcome data. Statistical heterogeneity among the included studies was analyzed by the *I*^2^ test. If there was no statistical heterogeneity between the studies (*P* > .05, *I*^2^ < 50%), the fixedeffect model was used for meta-analysis. If *P* ≤ .05 and *I*^2^ ≥ 50%, there is heterogeneity among the studies. We will use a sensitivity analysis to analyze the source of heterogeneity. After excluding the effect of heterogeneity, a random-effects model will be used for meta-analysis. A subgroup analysis will be performed for ulcerative colitis and Crohn disease.

## 3. Discussion

IBS-like symptoms, such as diarrhea, abdominal pain, and bloating, are often troubling quiescent IBD sufferers. And IBD has the characteristics of being easy to relapse, so patients have long-term severe psychological stress,^[15]^ especially in terms of diet. Most IBD patients are intolerant to lactose, fructose, and xylitol, which increases the burden of intestinal digestion, and residual short-chain sugars also stimulate intestinal flora.^[16]^ And lead to the expansion of small intestinal flora and the physical barrier of intestinal mucosal epithelium. It is obvious that The Low-FODMAP diet can reduce the burden of intestinal digestion and absorption, thereby improving gastrointestinal symptoms. Therefore, The Low-FODMAP diet has extensive application and promotion value, but there is still a lack of key evidence-based medical evidence, and its side effects are not yet clear. This study will provide evidence-based data for TFD treatment of IBD and provide new treatment options for future clinical applications.

## Acknowledgments

All the authors of this manuscript are very grateful to the various departments of Changchun University of Chinese Medicine for their support.

## Author contributions

Final approval of manuscript: All authors.

**Conceptualization:** Baijian Gu, Jianhua Zhou.

**Data curation:** Baijian Gu, Zhe Yu, Chengqiu Yan.

**Formal analysis:** Chong Shi, Chengqiu Yan.

**Funding acquisition:** Chong Shi.

**Investigation:** Baijian Gu, Bixin Chen.

**Methodology:** Baijian Gu, Jianhua Zhou.

**Project administration:** Zhe Yu, Jianhua Zhou.

**Software:** Bixin Chen.

**Supervision:** Zhe Yu, Jianhua Zhou.

**Validation:** Chengqiu Yan, Bixin Chen.

**Visualization:** Zhe Yu, Bixin Chen.

**Writing** - **original draft:** Baijian Gu, Zhe Yu, Chong Shi, Chengqiu Yan, Bixin Chen, Jianhua Zhou.

**Writing** - **review & editing:** Baijian Gu.
